# A Simple Approach to Bioconjugation at Diverse Levels: Metal-Free Click Reactions of Activated Alkynes with Native Groups of Biotargets without Prefunctionalization

**DOI:** 10.1155/2018/3152870

**Published:** 2018-12-12

**Authors:** Xianglong Hu, Xueqian Zhao, Benzhao He, Zheng Zhao, Zheng Zheng, Pengfei Zhang, Xiujuan Shi, Ryan T. K. Kwok, Jacky W. Y. Lam, Anjun Qin, Ben Zhong Tang

**Affiliations:** ^1^Department of Chemistry, Hong Kong Branch of Chinese National Engineering, Research Center for Tissue Restoration and Reconstruction, Institute of Advanced Study, State Key Laboratory of Molecular Nanoscience, Division of Life Science and Diversion of Biomedical Engineering, The Hong Kong University of Science and Technology, Clear Water Bay, Kowloon, Hong Kong, China; ^2^Ministry of Education Key Laboratory of Laser Life Science & Institute of Laser Life Science, College of Biophotonics, South China Normal University, Guangzhou 510631, China; ^3^HKUST-Shenzhen Research Institute, Shenzhen 518057, China; ^4^NSFC Center for Luminescence from Molecular Aggregates, SCUT-HKUST Joint Research Institute, State Key Laboratory of Luminescent Materials and Devices, South China University of Technology, Guangzhou 510640, China

## Abstract

The efficient bioconjugation of functional groups/molecules to targeted matrix and bio-related species drives the great development of material science and biomedicine, while the dilemma of metal catalysis, uneasy premodification, and limited reaction efficiency in traditional bioconjugation has restricted the booming development to some extent. Here, we provide a strategy for metal-free click bioconjugation at diverse levels based on activated alkynes. As a proof-of-concept, the abundant native groups including amine, thiol, and hydroxyl groups can directly react with activated alkynes without any modification in the absence of metal catalysis. Through this strategy, high-efficient modification and potential functionalization can be achieved for natural polysaccharide, biocompatible polyethylene glycol (PEG), synthetic polymers, cell penetrating peptide, protein, fast whole-cell mapping, and even quick differentiation and staining of Gram-positive bacteria, etc. Therefore, current metal-free click bioconjugation strategy based on activated alkynes is promising for the development of quick fluorescence labeling and functional modification of many targets and can be widely applied towards the fabrication of complex biomaterials and future* in vivo* labeling and detection.

## 1. Introduction

The structure and trafficking of biomolecules in biology and medicine is very attractive, but it is quite challenging to make it visible for researchers, which makes it hard for people to understand the biological process. For example, most biomolecules and synthetic biomaterials are essentially non-fluorescent; the most common bioconjugation is to tether a fluorescent dye or other imaging agents to the targeted species [1]. There are many potential candidates that need bioconjugation for biological research such as natural polysaccharide, protein, synthetic biofunctional polymers, biocompatible polyethylene glycol (PEG), colloidal particles, and even living organisms [2]. Bioconjugation chemistry is generally desirable for highly energetic reactants to form carbon-heteroatom (mostly N, O, and S) bonds. The bioconjugation reactions are expected to release no byproducts or release nitrogen or are condensation processes that produce water as the only byproducts [3, 4]. Typically, the diverse functions and structural properties of proteins make them hard to modify, thus limiting the downstream development in biology, chemistry, medicine, and many other disciplines. On the other hand, living organisms are even hard to be labeled covalently by general fluorescent dyes, which is usually achieved by complicated genetic technology. Herein, the complex diversity of these targets urgently calls for a widely applied methodology for the direct conjugation of targets without the need of prefunctionalization.

In the past several years, various synthetic strategies have been employed in bioconjugation chemistry, mainly containing two aspects; one is the conjugation based on the modification of extrinsic precursors on the species, followed by conjugation with the expected reporter or targeting units. Typically, the well-known copper-catalyzed azide-alkyne cycloaddition pioneered by Sharpless and Meldal et al. [5, 6] has emerged as a highly versatile bioconjugation strategy for tremendous targets, except in living cells and living organisms due to the toxicity of copper ions. Herein, much endeavor has been put to achieve metal-free process [7]. Furthermore, the premodification with azide or alkyne groups is not facile, possibly altering the primary function of biomolecules. Alternatively, the strain-promoted azide-alkyne cycloaddition, proposed by Bertozzi et al. [8–10], can avoid the employment of copper catalyst. The reactivity of the cyclooctyne can be modulated by tethering electron withdrawing groups at the propargylic position or by the increasing of strain energy through aryl ring or cyclopropyl ring fusion [11]. The optimized cyclooctyne has been demonstrated effectively in some mammalian cells and even animals, but the relatively big size and hydrophobic nature of the cyclooctyne units can significantly affect the distribution and biological properties of the species to which they are attached. The tedious synthesis of these cyclooctyne derivatives is also challenging and expensive, which is hard to approach for practical large-scale fabrication. Some other click reactions in this profile, such as Diels-Alder cycloadditions [12–14], tetrazine-norbornene reactions [15], and so on, are also facing the complex synthesis of precursors [16] or unfavorable UV light irradiation, which is restricted greatly for the wide application.

Another strategy is the direct conjugation of some functional units to some native groups in these biomolecule species without any previous modification [4]. These native groups are mainly composed by amine, thiol, and hydroxyl groups. Notably, amine and thiol groups in the side chain of amino acids are most widely employed for the modification of peptides and proteins [17]. Hydroxyl groups are popular in many polysaccharides that are abundant in biomedicine and living organisms [18]. Herein, this methodology has been extensively used in the modification of materials as well as the coupling of functional molecules in biological field due to its facile access and minimal influence to the activity of biomolecules.

On the other hand, the synthetic chemistry of alkynes has aroused our long-term interest in the past twenty years, especially focusing on alkynes-based polymer chemistry [19–23]. Among these, some metal-free click reactions have been developed based on alkynes and three kinds of native groups in biology, including amine, thiol, and hydroxyl groups. Specifically, spontaneous amino-yne click polymerization of dipropiolates and diamines was employed to prepare poly(enamine)s with 100% atom efficiency without any external catalyst in mild conditions [24]. Furthermore, catalyst-free thiol-yne click polymerization between dithiols and diynes was achieved in high yield, affording linear, and hyperbranched poly(vinylene sulfide)s, respectively [25–27]. In addition, hydroxyl groups can also react with alkynes in the presence of slight organic base, but not metal catalyst [28, 29].

**Figure 1:**
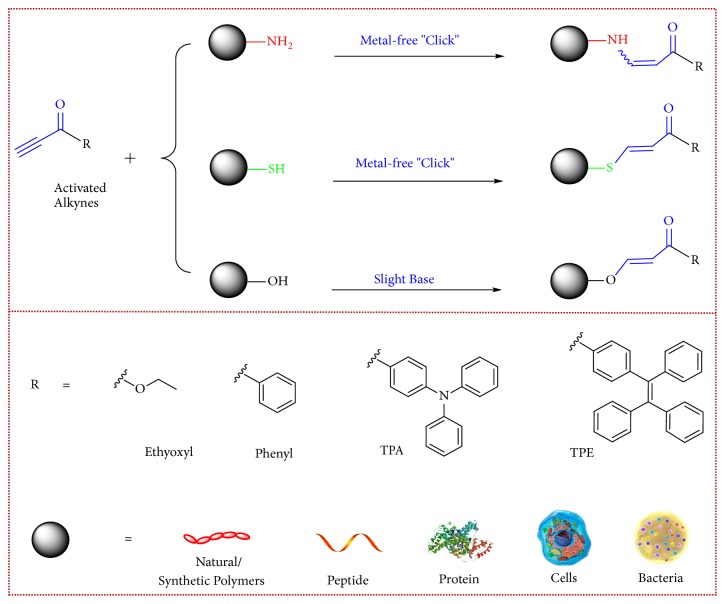
**Schematic illustration for the metal-free click bioconjugation with native groups of biotargets based on activated alkynes. **Native functional groups, including amine, thiol, and hydroxyl groups in different targets at diverse levels are employed to achieve on-demand modification of natural/synthetic polymers, peptide, protein, rapid whole cell mapping, and specific staining of Gram-positive bacteria.

Inspired by the research background of electron-deficient alkynes [30–32] and our previous work concerning activated alkynes, we envisage that these metal-free click reactions between optimized activated alkynes and the native groups will work for bioconjugation at diverse levels [33]. In this proof-of-concept study, metal-free on-demand click bioconjugation with natural polysaccharide, synthetic biofunctional polymers, peptides, proteins, further application for rapid whole cell mapping, and specific staining of Gram-positive bacteria were achieved (Figure 1). A series of activated alkynes with diverse substituent groups are examined for the fluorescence labeling of these potential candidates. The development of metal-free click bioconjugation based on activated alkynes is expected to expand the bioconjugation methodology and serve for material science and biomedicine in the future.

## 2. Results

### 2.1. Spontaneous Amino-Yne Click Bioconjugation for Chitosan and PEGylation

Four model activated alkyne compounds were examined in this work; ethyl propionate and ethynylcarbonylbenzene are commercially available; in addition,* N*-(4-ethynylcarbonylphenyl) diphenylamine (noted as* alkyne*-TPA) and* N*-(4-ethynylcarbonylphenyl) triphenylethylene (noted as* alkyne*-TPE) were readily prepared according to the reported literature ([Sec supplementary-material-1]) [34]; the molecular structure of* alkyne*-TPE was characterized accordingly ([Sec supplementary-material-1]-[Sec supplementary-material-1]). A model amino-yne click reaction between n-butylamine and ethynylcarbonylbenzene was examined at first ([Sec supplementary-material-1]a). The quantitative reaction finished at less than 30 min. Based on the ^1^H-NMR analysis, the resulting enamine product exhibited a mixture of* E*-and* Z*-configuration, in which the* Z* ratio was over ~92%, due to the probable intramolecular hydrogen bonding ([Sec supplementary-material-1]b) [35–37]. The resulting enamine product was further characterized by ^13^C-NMR and HRMS analysis ([Sec supplementary-material-1]). Furthermore, the amino-yne click reaction between amine and commercially available ethyl propiolate was also examined, which confirmed the fast click addition and the main* Z*-configuration for the *β*-aminoacrylate product ([Sec supplementary-material-1]).

**Figure 2:**
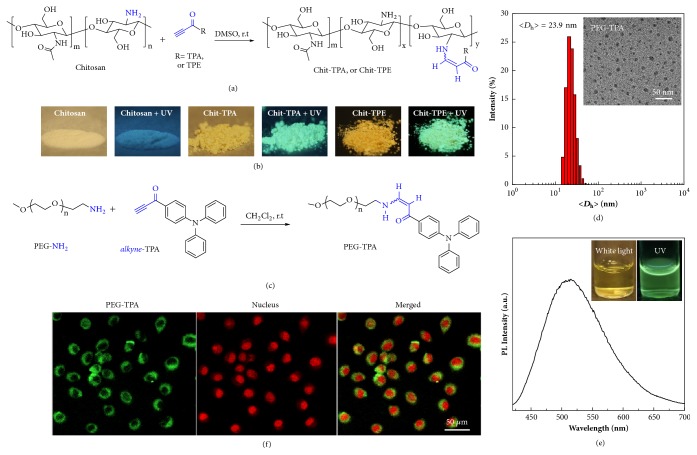
**Schematic illustration for metal-free amino-yne click bioconjugation between chitosan and PEG with fluorescent activated alkynes.** (a) Functionalization of natural polymers via the amino-yne click reaction. (b) Photographs of chitosan, Chit-TPA, and Chit-TPE in the presence of UV irradiation or not. (c) Synthetic rout of PEGylation of triphenylamine (TPA) via the amino-yne click reaction. (d) Hydrodynamic diameter distribution and TEM image (inset) determined for the aqueous dispersion of PEG-TPA. (e) Fluorescent emission spectrum of PEG-TPA aqueous dispersion (*λ*_ex_= 405 nm, insert photographs: the water dispersion under white light and UV irradiation). (f) CLSM images of EMT-6 cells upon incubating with the aqueous dispersion of PEG-TPA at 5 *μ*M for 4 h and cell nucleus were costained with Red dot1.

Then, a typical polysaccharide, chitosan, was selected to react with* alkyne*-TPA and* alkyne*-TPE, respectively (Figure 2(a)). Chitosan is a linear polysaccharide with many native amine groups at the side groups. After click bioconjugation, based on the FT-IR analysis, the representative stretching vibration of C≡C bond at ~2100 cm^−1^ disappeared in the resulting Chit-TPA and Chit-TPE, which both exhibited red-shifted solid fluorescence upon UV irradiation ([Sec supplementary-material-1], Figure 2(b)) [38]. The aqueous dispersion of Chit-TPA exhibited yellow color under white light and emitted green fluorescence under UV irradiation ([Sec supplementary-material-1]).

On the other hand, the covalent bioconjugation of polyethylene glycol (PEG) to a desired molecule is known as “PEGylation”, which is now a well-established method in biomedicine [39]. PEGylation mostly aims to enhance the blood retention duration of the therapeutics or imaging agents like proteins, enzymes, small molecular drugs, liposomes, and nanocarriers by protecting them against some degradation processes inside tissues or cells, which consequently optimizes the therapeutic outcome. As demonstrated, PEGylation effectively alters the pharmacokinetics of a variety of drugs and dramatically improves the pharmaceutical values [40–42]. Here, the amino-yne click reaction was demonstrated for the conjugation of fluorescent* alkyne*-TPA molecule with amine-terminated PEG, PEG-NH_2_ (Figure 2(c)). ^1^H-NMR analysis confirmed the complete reaction of terminal amine group with activated alkynes, exhibiting typical* Z*-configuration ratio to be ~92.6% versus ~7.4%* E*-configuration ([Sec supplementary-material-1]). The FT-IR characterization further demonstrated the total conversion of terminal alkyne and amine group ([Sec supplementary-material-1]).

In addition, PEG-NH_2_ was totally hydrophilic, but the conjugation of hydrophobic* alkyne*-TPA made the resultant PEG-TPA become amphiphilic. As shown in Figure 2(d), the aqueous self-assembly of PEG-TPA afforded narrowly dispersed nanoparticles, with the diameter determined to be ~23.9 nm by dynamic laser scattering (DLS) analysis. The nanoparticle size in dry state was slightly smaller than that in water due to the invisible nature of PEG under TEM analysis. Furthermore, the fluorescent emission spectrum of PEG-TPA aqueous dispersion exhibited typical green fluorescence (Figure 2(e)). Finally, the PEG-TPA water dispersion was employed to stain tumor cells for cell imaging (Figure 2(f)), upon incubation for 4 h; EMT-6 cells were obviously labeled by PEG-TPA. Thus, the metal-free click bioconjugation based PEGylation was expected to be promising for diverse applications.

### 2.2. Spontaneous Thiol-Yne Click Bioconjugation to Modify Synthetic Polymers

Another popular native group in many biotargets is thiol group. The model reaction between 1-hexanethiol and ethynylcarbonylbenzene was performed at first ([Sec supplementary-material-1]). The reaction was quickly finished in several minutes. The structure was characterized by ^1^H-NMR, ^13^C-NMR and MS analysis ([Sec supplementary-material-1]). ^1^H-NMR analysis proved the ~100%* E*-configuration. In addition, the model reaction between 1-hexanethiol and ethyl propionate was also examined ([Sec supplementary-material-1], [Sec supplementary-material-1]); the* E- *and* Z-*configuration are approximately 80% versus 20%. There was no intramolecular hydrogen bonding in the resulting vinylenesulfide product.

**Figure 3:**
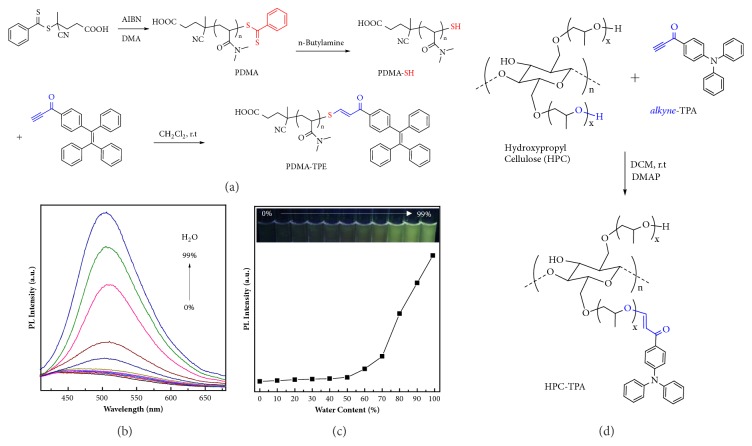
**Schematic illustration for metal-free thiol-yne and hydroxyl-yne reactions to modify synthetic polymer and polysaccharide. **(a) Modification of synthetic polymers derived from reversible addition-fragmentation chain-transfer (RAFT) polymerization. General synthetic route for the terminal modification with activated alkynes, as an illustration, affording PDMA-TPE. (b) Typical PL spectra of PDMA-TPE in water-THF mixtures with different water contents and (c) change of PL maximum of PDMA-TPE with water content of the aqueous mixture (insets: photographs of PDMA-TPE dispersion samples under UV illumination). (d) Synthetic route employed for the modification of hydroxypropyl cellulose (HPC) via the hydroxyl-yne reaction.

Native thiol groups are abundant in many biomolecules and potentially exist in some artificial species, such as synthetic polymers; specifically, synthetic polymers are increasing utilized as biomaterials in biomedicine. Typically, reversible addition fragmentation chain transfer (RAFT) polymerization is one of the most extensive polymerization methods to produce well-defined polymers. The end-group of RAFT agent can be facilelyconverted into a thiol group, providing opportunities for further modification [43, 44]. Herein, typical RAFT polymerization of* N,N*-dimethylacrylamide (DMA) and subsequent aminolysis afforded PDMA-SH (Figure 3(a)). The spontaneous thiol-yne click reaction between PDMA-SH and* alkyne*-TPE was performed to give PDMA-TPE. ^1^H-NMR analysis confirmed the modification efficiency up to 98.6% ([Sec supplementary-material-1]). After that, the aggregation-induced emission (AIE) characteristics of PDMA-TPE were investigated in water/THF mixtures with diverse water contents (*f*_W_), from 0 to 99% [45]. PDMA-TPE was almost non-emissive in THF solution in molecular state, mainly due to that the rotational motion of phenyl rings and double bonds in the terminal TPE unit was free to contribute nonradiative decay, leading to non-emission in solution state [46, 47]. The photoluminescence (PL) intensities gradually increased with the increase of water fraction due to the self-assembled aggregates of PDMA-TPE, exhibiting typical AIE-active profile (Figures 3(b) and 3(c)) [48]. Collectively, the thiol-yne click bioconjugation had the potency to modify bio-related synthetic polymers, and any biotargets with free thiol groups can be modified based on the thiol-yne click bioconjugation.

### 2.3. Metal-Free Hydroxyl-Yne Bioconjugation of Polysaccharide

Hydroxyl group is one of the most important native functionalities in biomolecules, including alcohols and sugars. As examined in our previous work, phenol-yne click polymerization could be achieved between activated diynes and diphenols to afford poly(vinylene ether ketone)s under mild reaction conditions, such as slight Lewis base catalyst, 4-dimethylaminopyridine (DMAP) [28]. If the alkynes were inactivated aromatic diynes, the polyhydroalkoxylation between aromatic diynes and alkyl diols would work in the presence of a superbase, strong phosphazene base (*t*-BuP_4_), finally affording poly(vinyl ether)s [29]. Currently, the model reaction between n-butyl alcohol and activated* alkyne*-TPA was examined ([Sec supplementary-material-1]a). The reaction could happen in the presence of slight DMAP to give ~100%* E*-configuration ([Sec supplementary-material-1]b, [Sec supplementary-material-1]).

Furthermore, natural polysaccharides are powerful candidates for biomedical engineering, because they are most potentially biocompatible, nontoxic, and renewable. Hydroxypropyl cellulose (HPC) is a representative derivative of natural polysaccharide cellulose with good water and organic solubility, in which some hydroxyl groups of cellulose have been hydroxypropylated to form propylene oxide groups [49, 50]. HPC has been approved by the United States Food and Drug Administration (FDA) and extensively applied in food and drug formulations. For a proof-of-concept, the fluorescence labeling of HPC by the activated alkynes was examined in the presence of slight DMAP (Figure 3(d)). As shown in [Sec supplementary-material-1], the conjugation product, HPC-TPA, displayed typical signals of phenyl protons in the ^1^H-NMR spectrum. The FT-IR spectrum of HPC-TPA revealed the disappearance of C≡C bond at ~2100 cm^−1^, whereas exhibiting typical stretching vibration of carbonyl groups ([Sec supplementary-material-1]). In spite of the participation of slight organic base in the hydroxyl-yne bioconjugation process, but thanks to the metal-free nature, it provides more opportunity for the conjugation of biomolecules for potential biological applications.

### 2.4. Metal-Free Click Fluorescence Labeling of Peptide and Protein

**Figure 4:**
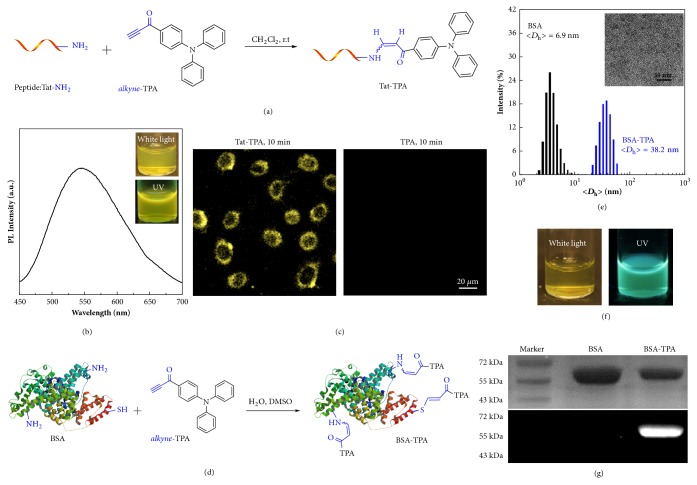
**Metal-free functionalization of peptide and protein.** (a) Schematic illustration for the thiol-yne click conjugation of Tat peptide with TPA, affording one cell-penetrating peptide conjugate, Tat-TPA. The sequence of Tat peptide is YGRKKRRQRRR. (b) Fluorescent emission spectrum of Tat-TPA (*λ*_ex_= 405 nm, insert photographs: Tat-TPA aqueous dispersion under white light and UV irradiation). (c) CLSM images of EMT-6 cells upon 30 min incubation with the aqueous dispersion of Tat-TPA and TPA at 2 *μ*M. No detectable fluorescence was observed for cells incubated with TPA. (d) Schematic illustration for the click conjugation of BSA with TPA, affording BSA conjugates, BSA-TPA. (e) Hydrodynamic diameter distribution of BSA and BSA-TPA in water (Inset: TEM image determined for BSA-TPA in water). (f) Photographs of BSA-TPA aqueous dispersion under white light and UV irradiation. (g) SDS-PAGE results recorded for BSA and BSA-TPA, and lower row image was taken under UV irradiation.

On the other hand, efficient on-demand modification of peptides and proteins is increasingly employed to provide synthetic vaccine candidates or target antigens in biomedicine. As demonstrated above, activated alkynes are able to react with three kinds of native groups including amine, thiol, and hydroxyl groups. Herein, current proof-of-concept methodology was examined to perform the fluorescence labeling of peptides and proteins. A Tat peptide (YGRKKRRQRRR) was employed to react with* alkyne*-TPA at ambient temperature in the absence of any catalysts (Figure 4(a)). Two amine side groups of lysine and the N-terminal amine group in the Tat peptide could efficiently conjugate with activated alkynes based on the above-mentioned procedure. The MALDI analysis of Tat and Tat-TPA showed that the difference of two molecular ion peaks was 297.88 ([Sec supplementary-material-1]), and the molecular weight of* alkyne*-TPA was 297.36; thus one TPA moiety was determined to be labeled in each Tat peptide chain. Although three amine groups in Tat were reactive, the resulting modification ratio was dependent on the initial equal molar feed ratio. The resulting Tat-TPA exhibited typical fluorescence emission at ~540 nm (Figure 4(b)). The cell-penetrating TAT peptide could facilitate efficient cellular internalization; thus HeLa cells were incubated with triphenylamine (TPA) and Tat-TPA for 10 min, respectively (Figure 4(c)). The cells treated with Tat-TPA were significantly fluorescent, while the group of TPA treating was almost non-fluorescent, which indirectly confirmed the successful conjugation of TPA with the Tat peptide.

On the other hand, bovine serum albumin (BSA) is a single polypeptide of ~66 kDa and consists of 59 lysine residues, of which 30-35 have primary amines that can be employed for bioconjugation. In addition, one non-oxidized cysteine (Cys-34) of BSA also provides one single thiol moiety for modification. Herein, native BSA was treated with* alkyne*-TPA in aqueous solution (Figure 4(d)). As expected, free amine groups and the free thiol moiety within BSA protein would react with* alkyne*-TPA to afford fluorescent TPA-labeled BSA, namely, BSA-TPA. The MALDI analysis was performed to estimate the conjugation efficiency of* alkyne*-TPA with BSA ([Sec supplementary-material-1]). Considering the contribution of quaternary amination of primary amine in BSA, ~3.98* alkyne*-TPA molecules were calculated to be labeled in each BSA protein. Furthermore, BSA was totally hydrophilic in water and existed as single molecule state, and TPA was hydrophobic, which made BSA-TPA amphiphilic accordingly. The hydrodynamic diameter of BSA was determined to be ~6.9 nm, whereas after bioconjugation with TPA, BSA-TPA exhibited a diameter of ~ 38.2 nm (Figure 4(e)). TEM was also employed to characterize the self-assembled morphology of BSA-TPA in water, exhibiting typical spherical nanoparticles with the diameter determined to be ~16 nm, which was smaller than the hydrodynamic size determined by DLS, because hydrophilic BSA coronas were invisible under TEM observation in the dry state. The aqueous dispersion exhibited yellow color under white light and emitted cyan color from TPA moieties (Figure 4(f)). Next, we investigated the formation of BSA-TPA bioconjugates by sodium dodecyl sulfate polyacrylamide gel electrophoresis (SDS-PAGE) (Figure 4(g)). SDS-PAGE results revealed the formation of fluorescent BSA-TPA conjugates in the second layer upon light irradiation, whereas native BSA was non-fluorescent. The molecular weight of BSA-TPA enhanced slightly compared with native BSA. Collectively, activated alkynes were able to functionalize peptides and proteins in aqueous solution without any catalysts.

### 2.5. Fast Whole-Cell Staining

**Figure 5:**
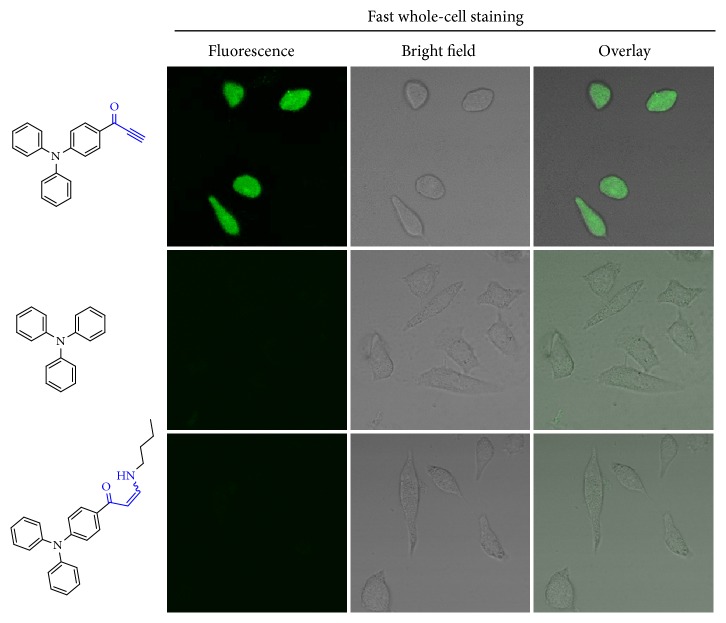
**Rapid whole-cell labeling and mapping of HeLa cells upon 2 min incubation with activated alkynes.** HeLa cells were quickly treated with three small fluorescent molecules, respectively, including TPA,* alkyne*-TPA, and the addition product of* alkyne*-TPA and n-butylamine.

The biological imaging has expanded intensively in recent years, but still faces many challenges, such as how to stain living cells quickly with improved spatial and temporal resolution for extended periods of staining time [51]. As a proof-of-concept, small molecular activated alkynes were examined for direct cell imaging (Figure 5). Three typical molecules including triphenylamine (TPA),* alkyne*-TPA, and the addition product of* alkyne*-TPA with* n*-butylamine were compared in parallel. For TPA, it does not possess activated triple bond and thus can not covalently conjugate with the substrate of biotargets. For the addition product of* alkyne*-TPA with* n*-butylamine, the triple bond has been reacted with primary amine already, which cannot further conjugate with amine group in physiological condition; therefore, for these three molecules, only* alkyne*-TPA is highly reactive towards primary amine and thiol species in biotargets. Herein, the comparison of these three molecules is expected to answer that whether activated alkynes are promising in biological imaging.

Upon incubation with HeLa cells for only 2 min in serum-free PBS media, remarkable fluorescence was found for cells treated with* alkyne*-TPA; more importantly, the whole cell region was fluorescent, whereas undetectable fluorescence was detected for two control molecules without activated alkynes or saturated with primary amine species. As demonstrated above,* alkyne*-TPA could react with primary amine group or thiol group quickly in the absence of any external catalyst. Herein, upon incubation with living cells in PBS,* alkyne*-TPA would probably conjugate with cellular species containing primary amine groups or thiol groups, such as amino acids, proteins, etc. in cell membrane as well as inside cells. The intracellular diffusion and trafficking of these conjugated fluorescent species would further promote the resulting fast whole-cell mapping. On the other hand, for two control molecules with similar main structure but without covalent potency in physiological condition, the cellular endocytosis or diffusion did not work well in so short incubation time [52, 53]. Collectively, we inclined to consider that the covalent bioconjugation of activated alkynes with cellular species played a dominant role for fast whole-cell staining. In-depth molecular biological analysis is underway to provide more evidence.

### 2.6. Quick Staining and Differentiation of Bacteria

**Figure 6:**
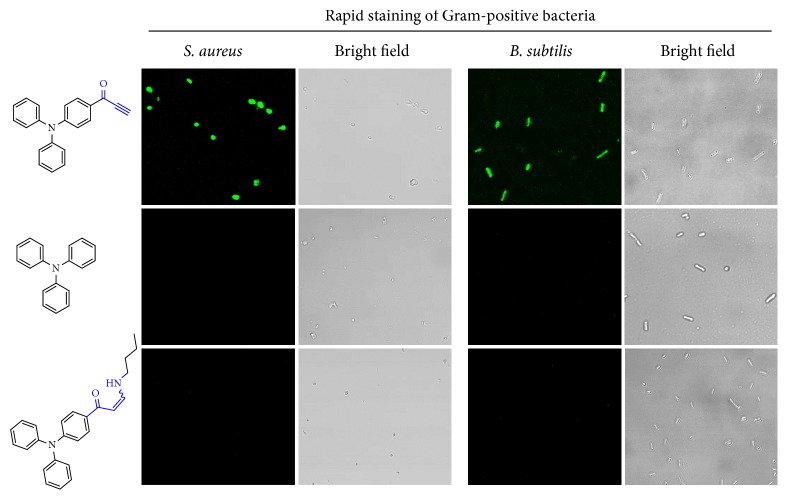
**Specific labeling of Gram-positive bacteria upon 2 min incubation with activated alkynes**. Two kinds of Gram-positive bacteria including* Staphylococcus aureus *(*S. aureus*) and* Bacillus subtilis* (*B. subtilis*) were incubated with three small fluorescent molecules, respectively, including TPA,* alkyne*-TPA, and the addition product of* alkyne*-TPA and n-butylamine.

Apart from fast whole-cell staining, activated alkynes were further examined for the imaging of living bacteria. Interestingly, Gram-positive bacteria (e.g.,* S. aureus *and* B. subtilis*) could be stained by* alkyne*-TPA after 2 min short incubation (Figure 6), but TPA and the addition product of* alkyne*-TPA with* n*-butylamine could not label these two types of Gram-positive bacteria, even after 30 min incubation; these two Gram-positive bacteria were hardly stained. Notably,* S. aureus *and* B. subtilis *are representative spherical and rod-like Gram-positive bacteria, respectively. On the other hand, two kinds of typical Gram-negative bacteria, including* E. coli* and* P. aeruginosa,* were also treated with these three fluorescent molecules for 10 min, respectively ([Sec supplementary-material-1]). Undetectable fluorescence was observed for all these three molecules. The main difference between Gram-positive bacteria and Gram-negative bacteria was the structural components of cell wall. Briefly, Gram-positive bacteria had a thick peptidoglycan layer (~20-80 nm), up to almost 90% of the whole cell wall, in which a protruding teichoic acid with free primary amine motif from alanine residues was attached to the outer phospholipid bilayer across the peptidoglycan layer [54]. Whereas, for Gram-negative bacteria, the teichoic acid component did not exist, the membrane was mainly composed by a thin peptidoglycan layer and phospholipid outer bilayer inserted with protruding lipopolysaccharide [55]. Thus, two probable reasons were inferred to contribute the activated alkynes-based quick differentiation and staining of Gram-positive bacteria from Gram-negative bacteria. The dominating reason was supposed to be the primary amine groups of typical teichoic acid in the membrane of Gram-positive bacteria; the primary amine could react with* alkyne*-TPA efficiently to label Gram-positive bacteria. Furthermore, much more complex outer membrane of Gram-negative bacteria possibly limited the facile and quick labeling compared with Gram-positive bacteria. It was inherently hard for exogenous species to enter into Gram-negative bacteria. Hence, current activated alkynes-based differentiation and staining of Gram-positive bacteria potentially benefited from efficient amino-yne click bioconjugation and the difference of bacteria membrane.

## 3. Discussion

Bioconjugation is an extremely important issue that can bridge biology, chemistry, and material science. Although great efforts have been made to promote bioconjugation chemistry forward, some key limitations of current available strategies have greatly restricted some profound applications in biology and biomedicine. Herein, a proof-of-concept methodology of metal-free click bioconjugation was proposed on the basis of activated alkynes. Three key advantages are demonstrated for this strategy. First, the metal-free feature highlights the great application in biology and medicine. Second, three kinds of abundant native groups, including amine, thiol, and hydroxyl groups in biotargets can be employed for facile labeling and functionalization, which can avoid the complexity and tedious premodification. Third, extensive biological species and potential applications can be achieved, including PEGylation, biofunctionalization of synthetic polymers, one-pot labeling of peptides and proteins, fast whole-cell mapping, and quick differentiation and labeling of Gram-positive bacteria.

On the other hand, facile site-specific bioconjugation in complex biological systems is one of the most challenging issues. The conjugation reactions between current activated alkynes with primary amine and thiol groups are both quick and sufficient in mild conditions, exhibiting limited selectivity. However, the labeling content is generally very low in many cases, which is enough for subsequent applications, and thus the precise labeling location is not so imperative in these systems. In addition, the proposed metal-free process in this work is further expanded to functionalize inorganic surface ([Sec supplementary-material-1]). Silicon particles were facilely labeled with blue TPE or green TPA via the amino-yne click reaction, exhibiting blue and green fluorescence, respectively. Herein, metal-free bioconjugation based on activated alkynes not only provides a general platform for facile biocompatible labeling of biotargets, but also a general methodology for the efficient modification of both organic and inorganic materials, definitely exhibiting broad appeal to biology, chemistry, and material science. The exploration of activated alkynes in bioorthogonal chemistry with high selectivity is our ongoing target, and the* in vivo *theranostic application of this strategy is also underway.

## 4. Materials and Methods

### 4.1. Materials

*N,N*-dimethylacrylamide (DMA), amine-terminated polyethylene glycol (PEG-NH_2_), hydroxypropyl cellulose (HPC), bovine serum albumin (BSA), triphenylamine (TPA), and ethyl propiolate were obtained from Aldrich and used as received without further purification. 2,2'-azobis(2- methylpropionitrile) (AIBN) was obtained from Acros chemicals and recrystallized from 95% ethanol.* N*-(4-ethynylcarbonylphenyl) diphenylamine (*alkyne*-TPA) was prepared as previously [34], which was also available from AIE_GEN_ Biotech Co., Ltd. All solvents were purchased from Aldrich and used as received without further purification.

^1^H NMR and ^13^C NMR spectra were measured on a Bruker ARX 300 spectrometer using tetramethylsilane as internal reference. FT-IR spectra were taken on a Nicolet iS50 spectrometer. Photoluminescence (PL) spectra were recorded on a Perkin-Elmer LS55 spectrofluorometer. TEM measurements were conducted on a JEOL 2010 electron microscope. The sample for TEM observations was prepared by placing 20 *μ*L water dispersion on copper grids, followed by natural drying without negative staining operation.

### 4.2. Synthesis of TPA/TPE-Labeled Chitosan

Chitosan was dissolved in aqueous solution; then the DMSO solution of* alkyne*-TPA or* alkyne*-TPE was added in the above solution, respectively. After 10 h stirring, the solution was dialyzed against water and freeze-dried to afford Chit-TPA and Chit-TPE, respectively.

### 4.3. Synthesis of PEG-TPA

Amine-terminated PEG (PEG-NH_2_) was dissolved in dichloromethane; then equal molar ratio of* alkyne*-TPA was added into the flask. After stirring at room temperature for 1 h, the mixture was precipitated into an excess of diethyl ether to generate solid residues; the residues were dissolved in dichloromethane and precipitated into diethyl ether, which was repeated for three times. The final product was dried in a vacuum oven overnight at room temperature, affording PEG-TPA.

### 4.4. Synthesis of PDMA-TPE

Typical reversible addition-fragmentation chain transfer (RAFT) polymerization was employed for the synthesis of PDMA hydrophilic polymer [56]. Chain transfer agent, DMA, and AIBN were charged into a glass ampoule containing 1,4-dioxane. The ampoule was then degassed via three freeze-pump-thaw cycles and flame-sealed under vacuum. Then it was immersed into an oil bath thermostated at 70°C to start the polymerization. After 12 h, the ampoule was quenched into liquid nitrogen. The mixture was precipitated into an excess of diethyl ether to give light pink residues; the residues were dissolved in dichloromethane and precipitated into diethyl ether. This process was performed three times to give PDMA ([Sec supplementary-material-1]a). Then, four-molar ratio of n-butylamine was employed to treat PDMA in dichloromethane; affording PDMA terminated with one thiol group, PDMA-SH, the removal of phenyl group was characterized by ^1^H NMR spectrum ([Sec supplementary-material-1]b). After that, PEG-SH and* alkyne*-TPE were mixed with the same molar ratio in dichloromethane, and the reaction was stopped after 1 h stirring. The mixture was precipitated into an excess of diethyl ether to afford the resultant product, PDMA-TPE, and demonstrated by ^1^H NMR spectrum ([Sec supplementary-material-1]c).

### 4.5. Synthesis of HPC-TPA

HPC and* alkyne*-TPA were dissolved in dichloromethane and kept stirring overnight. The mixture was precipitated into an excess of diethyl ether to give solid residues, and the residues were dissolved in dichloromethane and precipitated into diethyl ether, which was repeated for three times. The resultant HPC-TPA was characterized by ^1^H NMR spectrum ([Sec supplementary-material-1]).

### 4.6. Fluorescent Bioconjugation of Peptides and Proteins

For the fluorescent labeling of Tat peptide, Tat was dissolved in dichloromethane; then equal molar ratio of* alkyne*-TPA was added into the flask. After stirring for several hours at room temperature, the resulting Tat-TPA was fabricated via typical three cycles of precipitation and dissolution. Similarly, BSA was dissolved in water and then added with the DMSO solution of* alkyne*-TPA; upon stirring, the dispersion was dialyzed against water overnight and then freeze-dried to afford BSA-TPA.

### 4.7. Cell Culture and Imaging

HeLa cells and EMT-6 cells were cultured in Dulbecco's modified Eagle's medium (DMEM), respectively. The media was supplemented with 10% fetal bovine serum (FBS) and 1% penicillin−streptomycin in 5% CO_2_ at 37°C in a humidified incubator. Before each experiment, the cells were precultured until confluence was reached. For the examination of cell imaging potency for PEG-TPA, EMT-6 cells were cultured for 4 h and then imaged by a commercial laser scanning microscope (LSM 510/ConfoCor 2, Zeiss, Jena, Germany). The fluorescent channel was recorded at the excitation wavelength of 405 nm. Secondly, for the imaging by Tat-TPA against EMT-6 cells, EMT-6 cells were incubated with Tat-TPA and TPA for 10 min, respectively, and then imaged by CLSM. Finally, for the imaging potency of three kinds of three small molecules, TPA,* alkyne*-TPA, and the conjugation product of* alkyne*-TPA with n-butylamine, these three molecules were treated with HeLa cells for 2 min in PBS, then washed with cold PBS, and imaged immediately by the CLSM system, respectively.

### 4.8. Bacteria Identification and Staining

Bacteria dispersion (10^7^ CFU/mL, 200 *μ*L) was incubated with TPA,* alkyne*-TPA, and the conjugation product of* alkyne*-TPA with n-butylamine (2 *μ*M) in PBS at room temperature for predetermined durations. Subsequently, the resultant mixtures were centrifuged at 8000 rpm for 3 min to remove the supernatant and washed with PBS for three times. The bacterial pellet was resuspended into PBS and imaged by the CLSM system.

## Data Availability

Supporting Information is available from the website or from the author.

## Supplementary Materials

Supplementary Materials
** Figure S1**. Schematic illustration for the synthesis of alkyne-functionalized triphenylamine,* alkyne*-TPA, and alkyne-functionalized tetraphenylethylene,* alkyne*-TPE.** Figure S2**. ^1^H NMR spectrum recorded for* alkyne*-TPE in CDCl_3_.** Figure S3**. ^13^C NMR spectrum recorded for* alkyne*-TPE in CDCl_3_.** Figure S4**. HRMS result recorded for* alkyne*-TPE.** Figure S5**. (a) Synthetic route of the model reaction between butylamine and ethynylcarbonylbenzene in a metal-free mild profile and (b) ^1^H-NMR spectrum recorded in CDCl_3_ for the precursor and the resultant product.** Figure S6**. (a) ^13^C-NMR spectrum and (b) HRMS result obtained for the conjugation molecule between butylamine and ethynylcarbonylbenzene.** Figure S7**. (a) Synthetic route of the model reaction between ethylamine and ethyl propionate in a metal-free mild profile, (b) ^1^H-NMR spectrum, and (c) ^13^C-NMR spectrum recorded for the resultant product.** Figure S8**. FT-IR spectra recorded for the conjugation between chitosan and* alkyne*-TPA,* alkyne*-TPE to afford Chit-TPA, and Chit-TPE, respectively.** Figure S9**. Fluorescent emission spectrum obtained for the aqueous dispersion of Chit-TPA, insets: photographs of the water dispersion under room light and UV irradiation.** Figure S10**. ^1^H-NMR spectra recorded in CDCl_3_ for* alkyne*-TPA, PEG-NH_2_, and the conjugation product, PEG-TPA.** Figure S11**. FT-IR spectra recorded for* alkyne*-TPA, PEG-NH_2_, and PEG-TPA.** Figure S12**. (a) The model reaction between 1-hexanethiol and ethynylcarbonylbenzene in a metal-free profile, (b) ^1^H-NMR, and (c) ^13^C-NMR spectra recorded for the resultant product.** Figure S13**. HRMS result for the metal-free conjugation product between 1-hexanethiol and ethynylcarbonylbenzene.** Figure S14**. The model reaction between 1-hexanethiol and ethyl propionate in a metal-free profile and ^1^H-NMR spectrum recorded for the conjugation product in CDCl_3_.** Figure S15**. HRMS result for the metal-free conjugation product between1-hexanethiol and ethyl propionate.** Figure S16**. ^1^H-NMR spectra recorded for PDMA, PDMA-SH, and PDMA-TPE in CDCl_3_**. Figure S17**. (a) The model reaction between 1-butanol and* alkyne*-TPA in the presence of slight DMAP, (b) ^1^H-NMR, and (c) ^13^C-NMR spectra recorded for the conjugation product.** Figure S18**. HRMS result for the conjugation product between 1-butanol and* alkyne*-TPA.** Figure S19**. ^1^H-NMR spectra recorded for HPC and the conjugation product of HPC and* alkyne*-TPA, HPC-TPA.** Figure S20**. FT-IR spectra recorded for the conjugation product of* alkyne*-TPA and HPC, HPC-TPA, respectively.** Figure S21**. MALDI TOF analysis of Tat peptide and* alkyne*-TPA functionalized Tat, Tat-TPA, respectively. The sequence of Tat peptide is YGRKKRRQRRR.** Figure S22**. MALDI TOF analysis of BSA and* alkyne*-TPA functionalized BSA, BSA-TPA, respectively.** Figure S23**. Two types of typical Gram-negative bacteria,* Escherichia coli* (*E. coli*) and* Pseudomonas aeruginosa *(*P. aeruginosa*), upon 10 min incubation with three kinds of fluorescent molecules, respectively, including TPA,* alkyne*-TPA, and the addition product of* alkyne*-TPA and n-butylamine.** Figure S24**. Preliminary demonstration for the functionalization of inorganic matrix (such as SiO_2_ particles) via the amino-yne click conjugation. (a) Schematics for catalyst-free fabrication process, affording TPE and TPA-modified SiO_2_ particles, SiO_2_-TPA, SiO_2_-TPE, respectively. (b) Fluorescent microscopy imaging of SiO_2_-TPA, SiO_2_-TPE, respectively (*λ*_ex_= 365 nm).
