# Supplementary material for: A Simple Approach to Bioconjugation at Diverse Levels: Metal-Free Click Reactions of Activated Alkynes with Native Groups of Biotargets without Prefunctionalization
Source: Research (Wash D C). 2018 Dec 12;2018:3152870. doi: 10.1155/2018/3152870 (PMC6750040; doi:10.1155/2018/3152870)
Supplement: Supplementary Materials — Figure S1. Schematic illustration for the synthesis of alkyne-functionalized triphenylamine, alkyne-TPA, and alkyne-functionalized tetraphenylethylene, alkyne-TPE. Figure S2. 1H NMR spectrum recorded for alkyne-TPE in CDCl3. Figure S3. 13C NMR spectrum recorded for alkyne-TPE in CDCl3. Figure S4. HRMS result recorded for alkyne-TPE. Figure S5. (a) Synthetic route of the model reaction between butylamine and ethynylcarbonylbenzene in a metal-free mild profile and (b) 1H-NMR spectrum recorded in CDCl3 for the precursor and the resultant product. Figure S6. (a) 13C-NMR spectrum and (b) HRMS result obtained for the conjugation molecule between butylamine and ethynylcarbonylbenzene. Figure S7. (a) Synthetic route of the model reaction between ethylamine and ethyl propionate in a metal-free mild profile, (b) 1H-NMR spectrum, and (c) 13C-NMR spectrum recorded for the resultant product. Figure S8. FT-IR spectra recorded for the conjugation between chitosan and alkyne-TPA, alkyne-TPE to afford Chit-TPA, and Chit-TPE, respectively. Figure S9. Fluorescent emission spectrum obtained for the aqueous dispersion of Chit-TPA, insets: photographs of the water dispersion under room light and UV irradiation. Figure S10. 1H-NMR spectra recorded in CDCl3 for alkyne-TPA, PEG-NH2, and the conjugation product, PEG-TPA. Figure S11. FT-IR spectra recorded for alkyne-TPA, PEG-NH2, and PEG-TPA. Figure S12. (a) The model reaction between 1-hexanethiol and ethynylcarbonylbenzene in a metal-free profile, (b) 1H-NMR, and (c) 13C-NMR spectra recorded for the resultant product. Figure S13. HRMS result for the metal-free conjugation product between 1-hexanethiol and ethynylcarbonylbenzene. Figure S14. The model reaction between 1-hexanethiol and ethyl propionate in a metal-free profile and 1H-NMR spectrum recorded for the conjugation product in CDCl3. Figure S15. HRMS result for the metal-free conjugation product between1-hexanethiol and ethyl propionate. Figure S16. 1H-NMR spectra recorded for PDMA, [file 3152870.f1.docx]

Supplementary Materials

A Simple Approach to Bioconjugation at Diverse Levels: Metal-Free Click Reactions of Activated Alkynes with Native Groups of Biotargets without Prefunctionalization

**Xianglong Hu,^1,2^ Xueqian Zhao,^1^ Benzhao He,^1^ Zheng Zhao,^1^** **Zheng Zheng,^1^ Pengfei Zhang,^1^ Xiujuan Shi,^1,3^ Ryan T. K. Kwok,^1^ Jacky W. Y. Lam,^1^ Anjun Qin,^4^ and Ben Zhong Tang^1,3,4,^***

*^1^* *Department of Chemistry, Hong Kong Branch of Chinese National Engineering, Research Center for Tissue Restoration and Reconstruction, Institute of Advanced Study, State Key Laboratory of Molecular Nanoscience, Division of Life Science and Diversion of Biomedical Engineering, The Hong Kong University of Science and Technology, Clear Water Bay, Kowloon, Hong Kong, China.*

*^2^Ministry of Education Key Laboratory of Laser Life Science & Institute of Laser Life Science, College of Biophotonics, South China Normal University, Guangzhou, 510631, China.*

*^3^ HKUST-Shenzhen Research Institute, Shenzhen 518057, China.*

*^4^**NSFC Center for Luminescence from Molecular Aggregates, SCUT-HKUST Joint Research Institute, State Key La-boratory of Luminescent Materials and Devices, South China University of Technology, Guangzhou 510640, China.*

***** Correspondence should be addressed to Ben Zhong Tang; tangbenz@ust.hk

**Supplementary Figures:**

**Figure S1**. Schematic illustration for the synthesis of alkyne-functionalized triphenylamine, *alkyne*-TPA, and alkyne-functionalized tetraphenylethylene, *alkyne*-TPE.


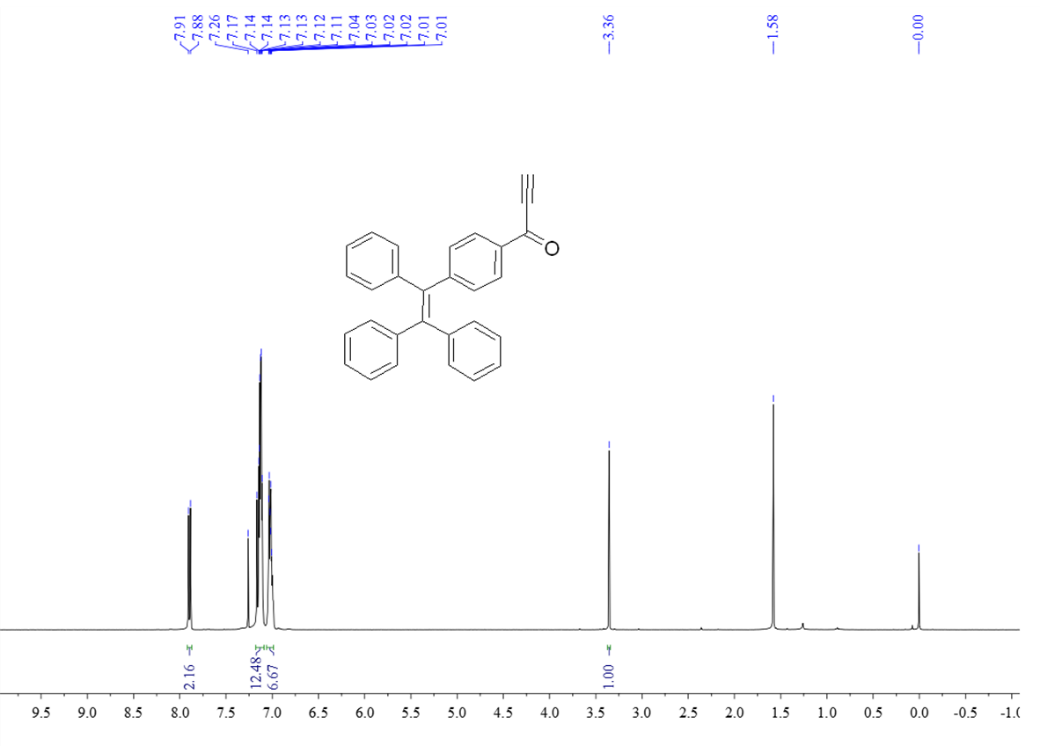


**Figure S2**. ^1^H NMR spectrum recorded for *alkyne*-TPE in CDCl_3_.


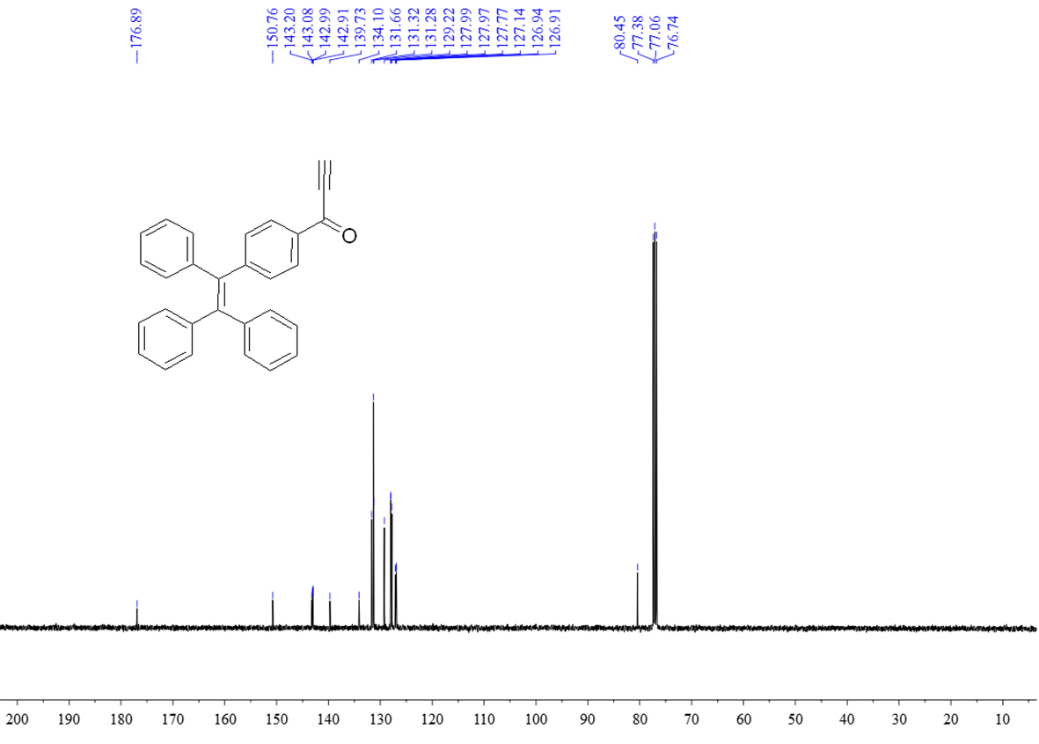


**Figure S3**. ^13^C NMR spectrum recorded for *alkyne*-TPE in CDCl_3_.


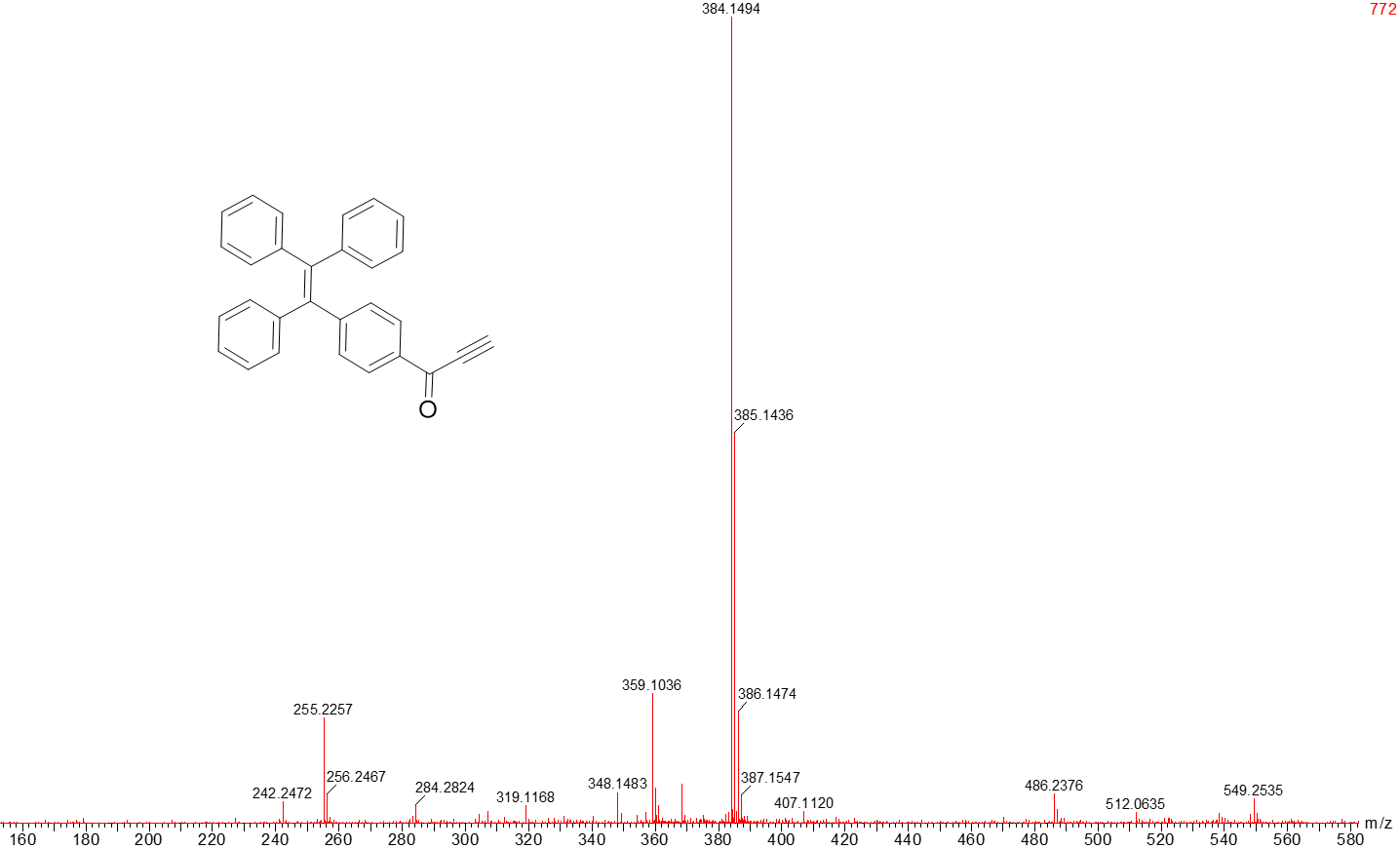


**Figure S4**. HRMS result recorded for *alkyne*-TPE.


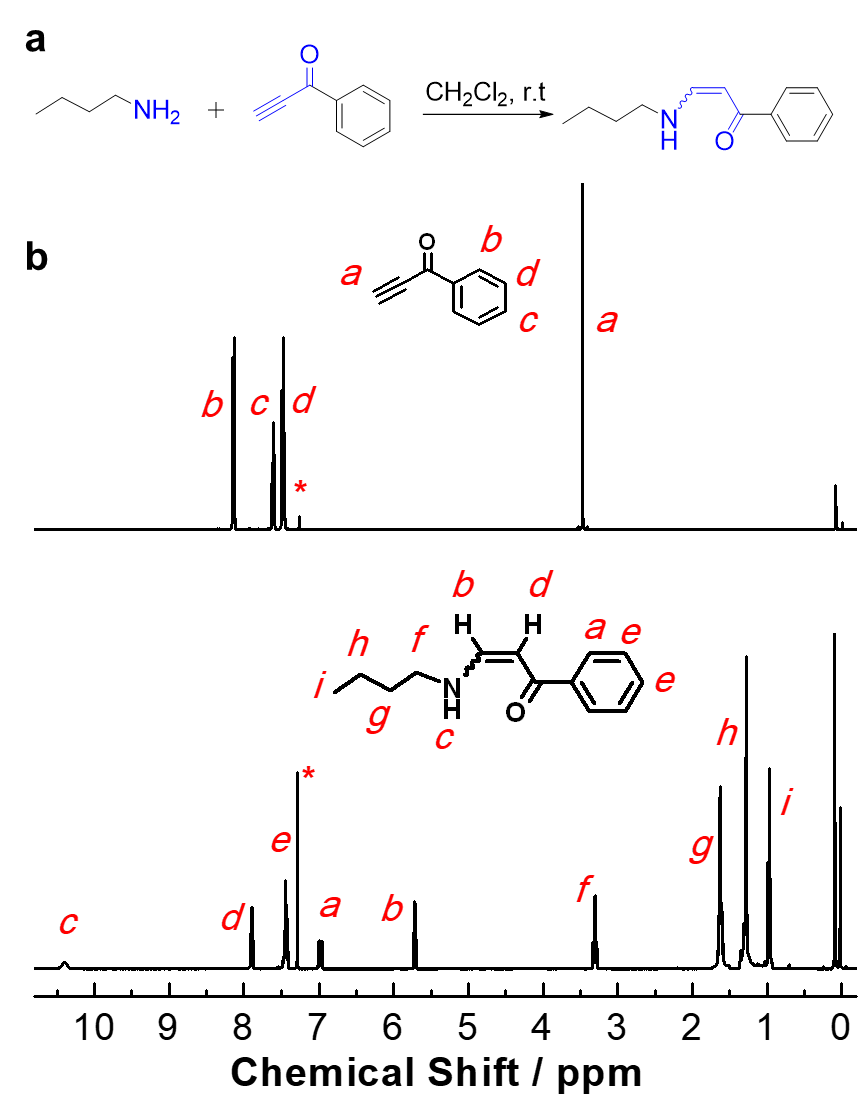


**Figure S5**. (a) Synthetic route of the model reaction between butylamine and ethynylcarbonylbenzene in a metal-free mild profile, and (b) ^1^H-NMR spectrum recorded in CDCl_3_ for the precursor and the resultant product.


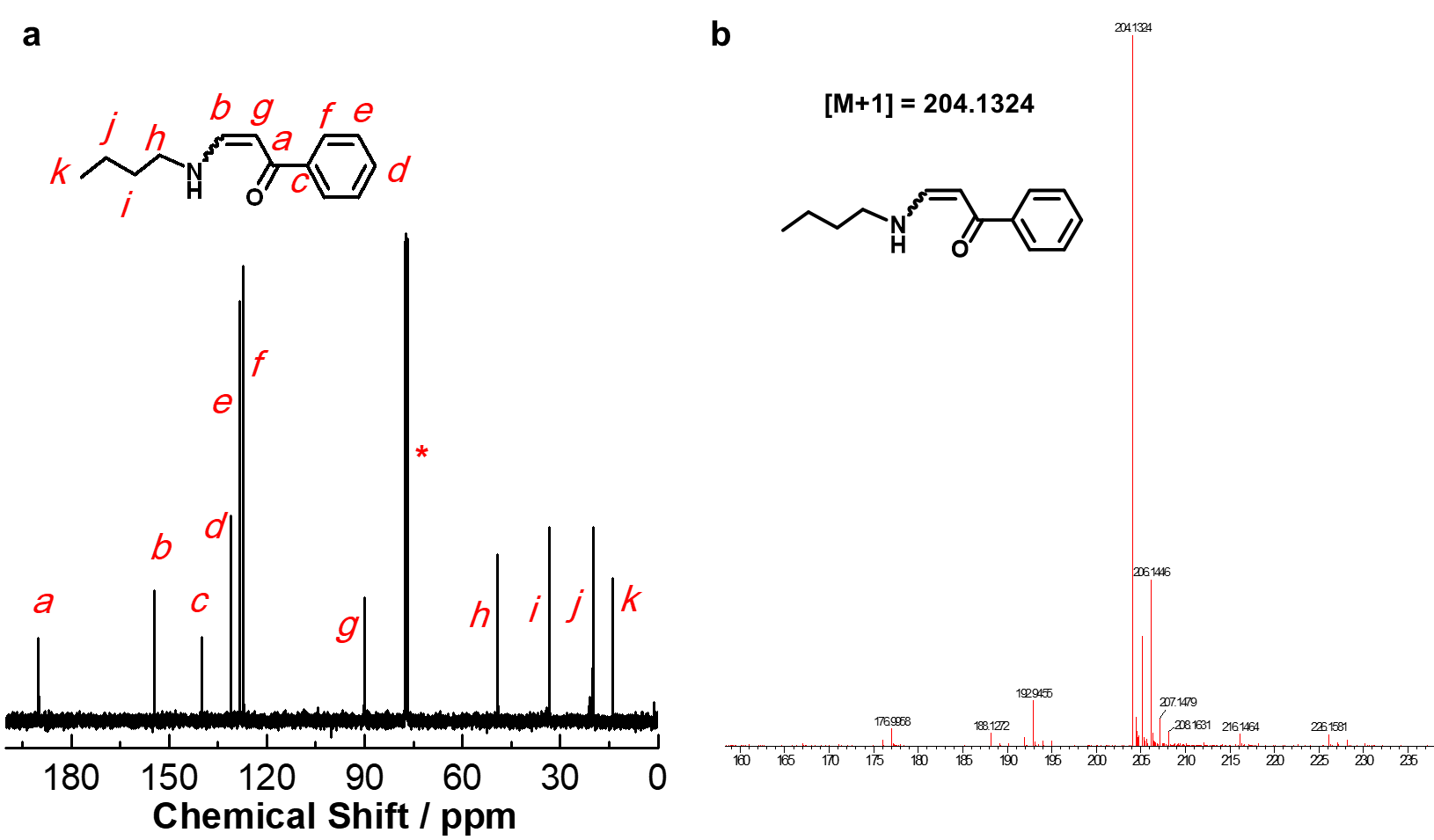


**Figure S6**. (a) ^13^C-NMR spectrum and (b) HRMS result obtained for the conjugation molecule between butylamine and ethynylcarbonylbenzene.


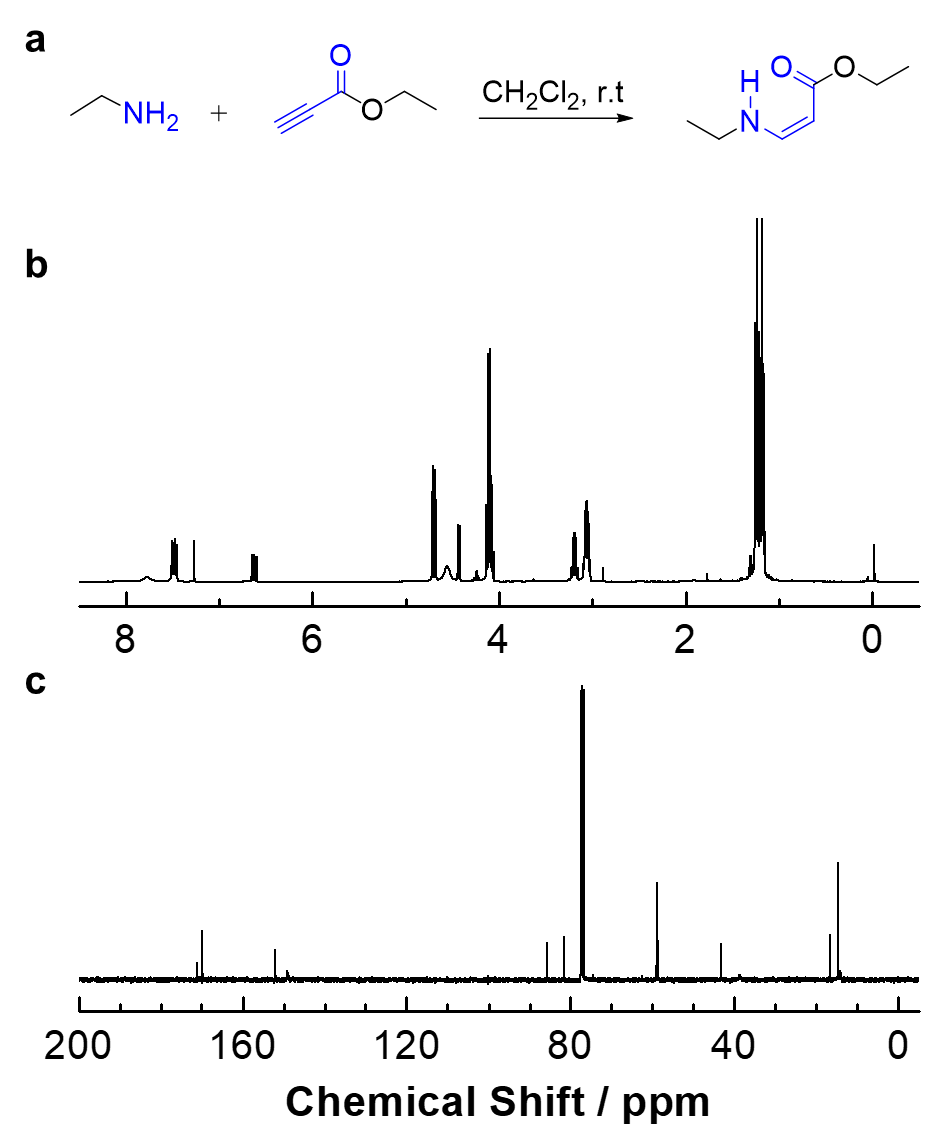


**Figure S7**. (a) Synthetic route of the model reaction between ethylamine and ethyl propionate in a metal-free mild profile, (b) ^1^H-NMR spectrum and (c) ^13^C-NMR spectrum recorded for the resultant product.

**Figure S8**. FT-IR spectra recorded for the conjugation between chitosan and *alkyne*-TPA, *alkyne*-TPE to afford Chit-TPA and Chit-TPE, respectively.


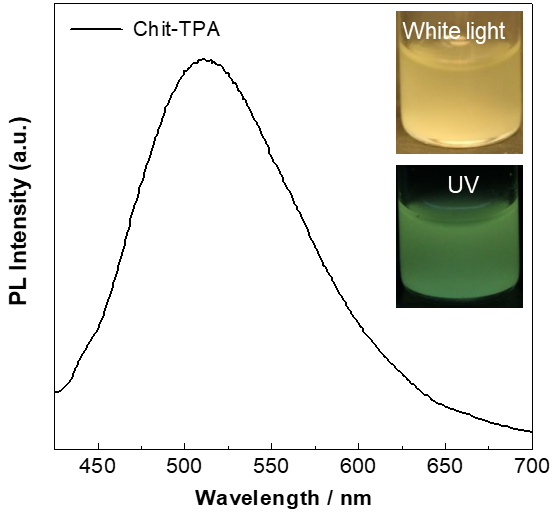


**Figure S9**. Fluorecent emission spectrum obtained for the aqueous dispersion of Chit-TPA, insets: photographs of the water dispersion under room light and UV irradiation.

**Figure S10**. ^1^H-NMR spectra recorded in CDCl_3_ for *alkyne*-TPA, PEG-NH_2_, and the conjugation product, PEG-TPA.

**Figure S11**. FT-IR spectra recorded for *alkyne*-TPA, PEG-NH_2_, and PEG-TPA.


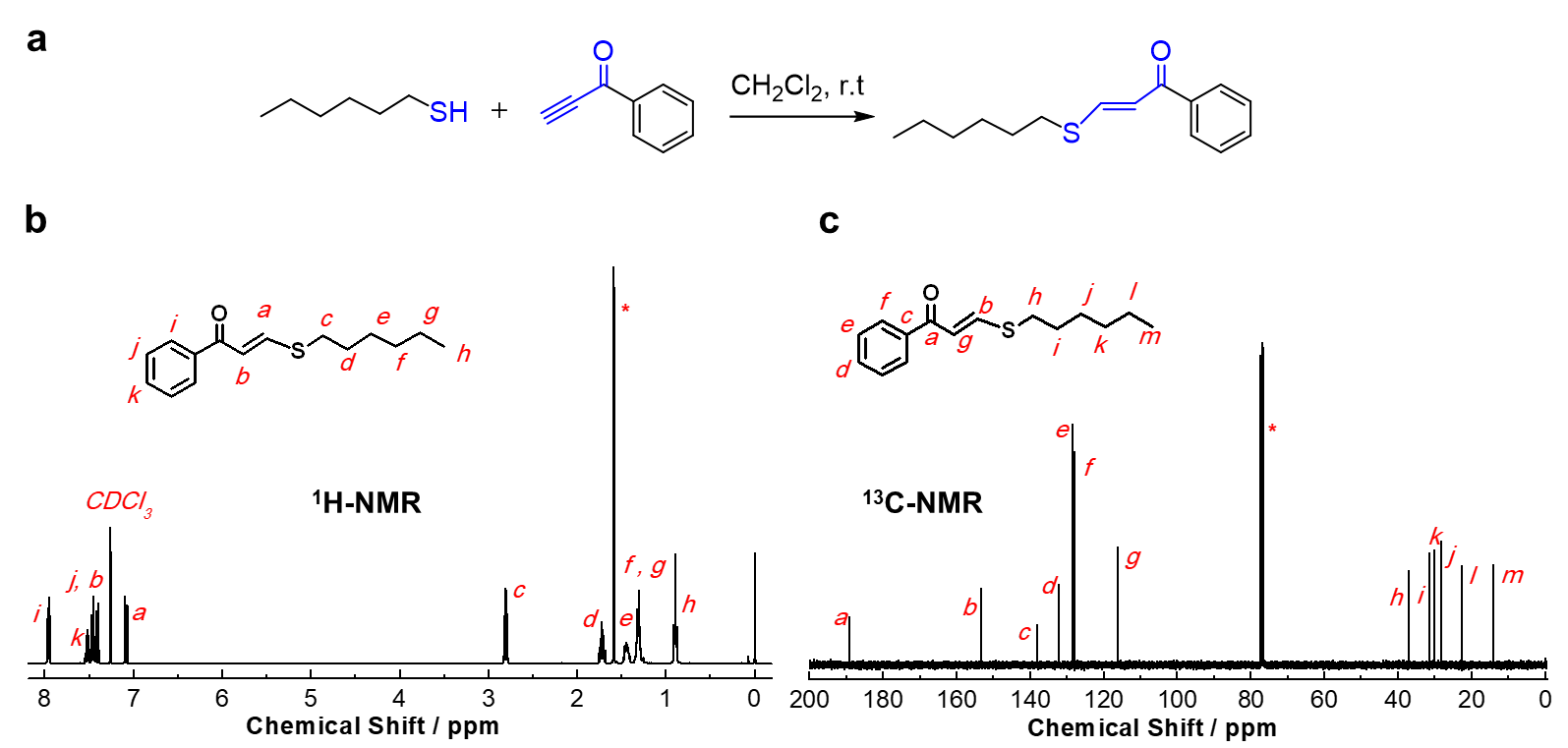


**Figure S12**. (a) The model reaction between 1-hexanethiol and ethynylcarbonylbenzene in a metal-free profile, (b) ^1^H-NMR and (c) ^13^C-NMR spectra recorded for the resultant product.

\
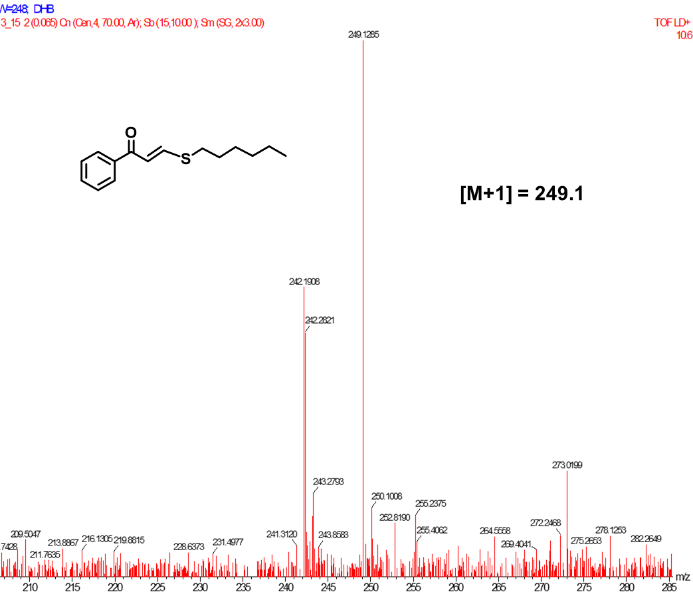


**Figure S13**. HRMS result for the metal-free conjugation product between 1-hexanethiol and ethynylcarbonylbenzene.


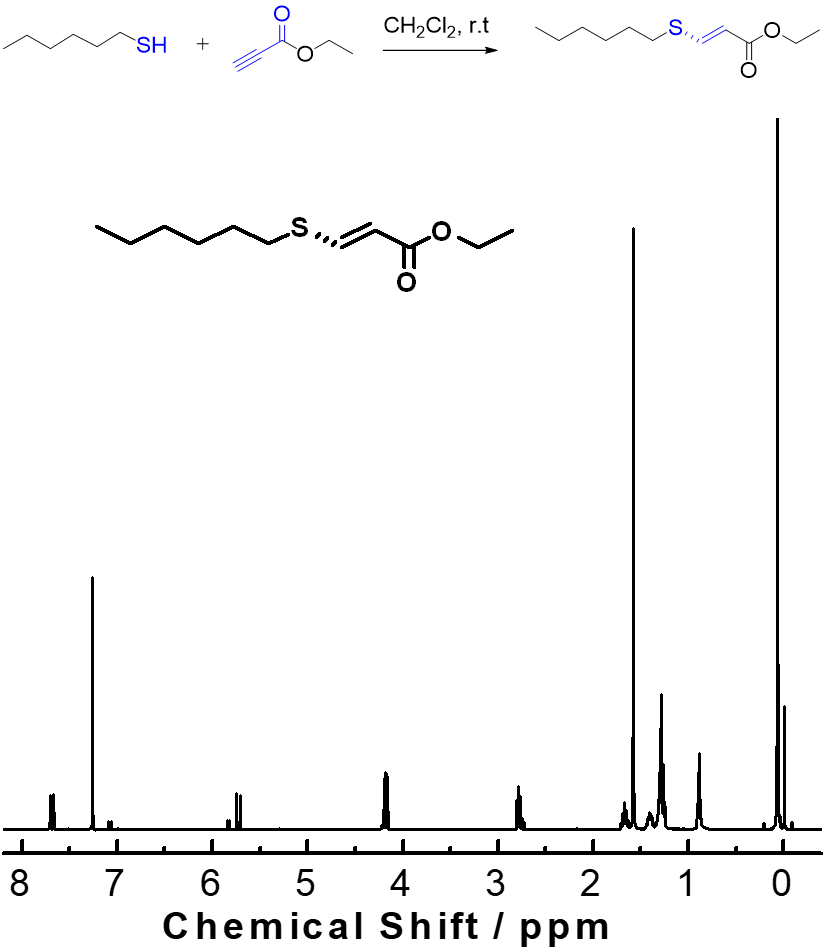


**Figure S14**. The model reaction between 1-hexanethiol and ethyl propionate in a metal-free profile and ^1^H-NMR spectrum recorded for the conjugation product in CDCl_3_.


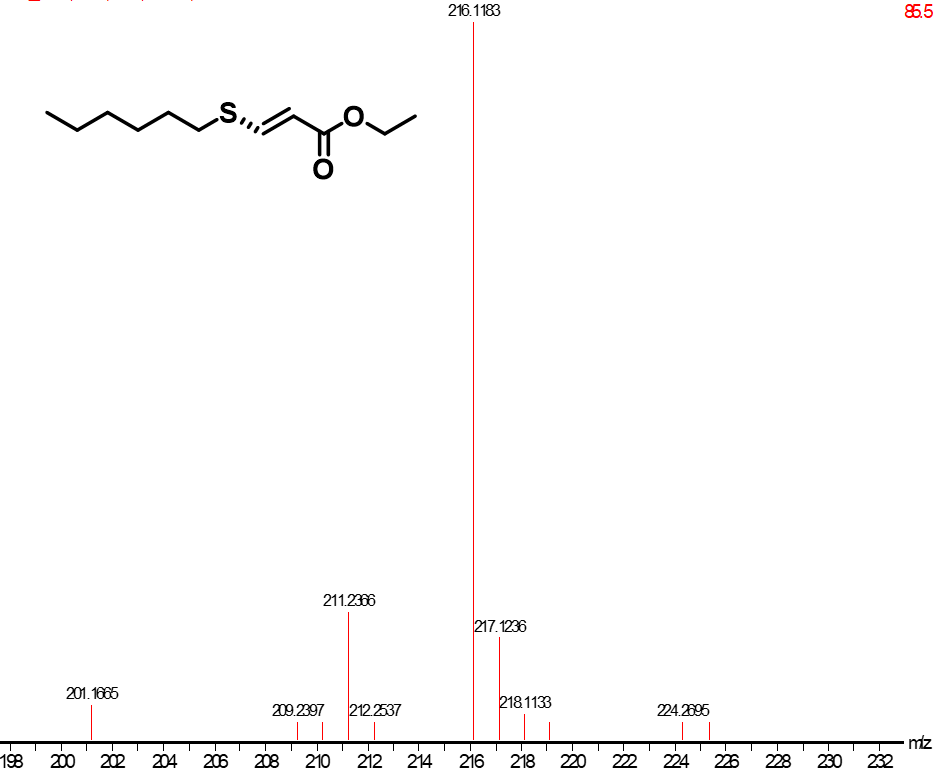


**Figure S15**. HRMS result for the metal-free conjugation product between1-hexanethiol and ethyl propionate.

**Figure S16**. ^1^H-NMR spectra recorded for PDMA, PDMA-SH, and PDMA-TPE in CDCl_3_.


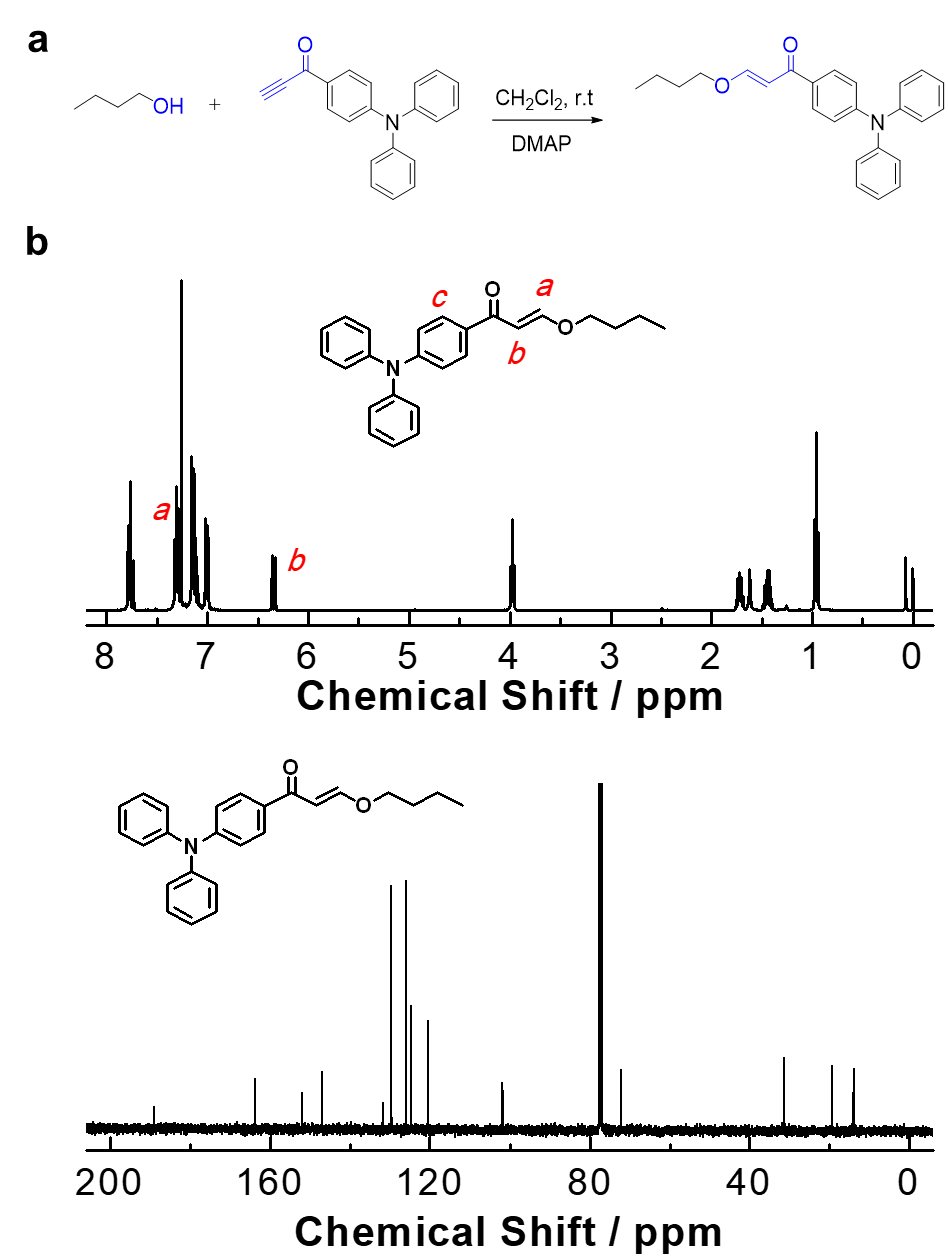


**Figure S17**. (a) The model reaction between 1-butanol and *alkyne*-TPA in the presence of slight DMAP, (b) ^1^H-NMR and (c) ^13^C-NMR spectra recorded for the conjugation product.


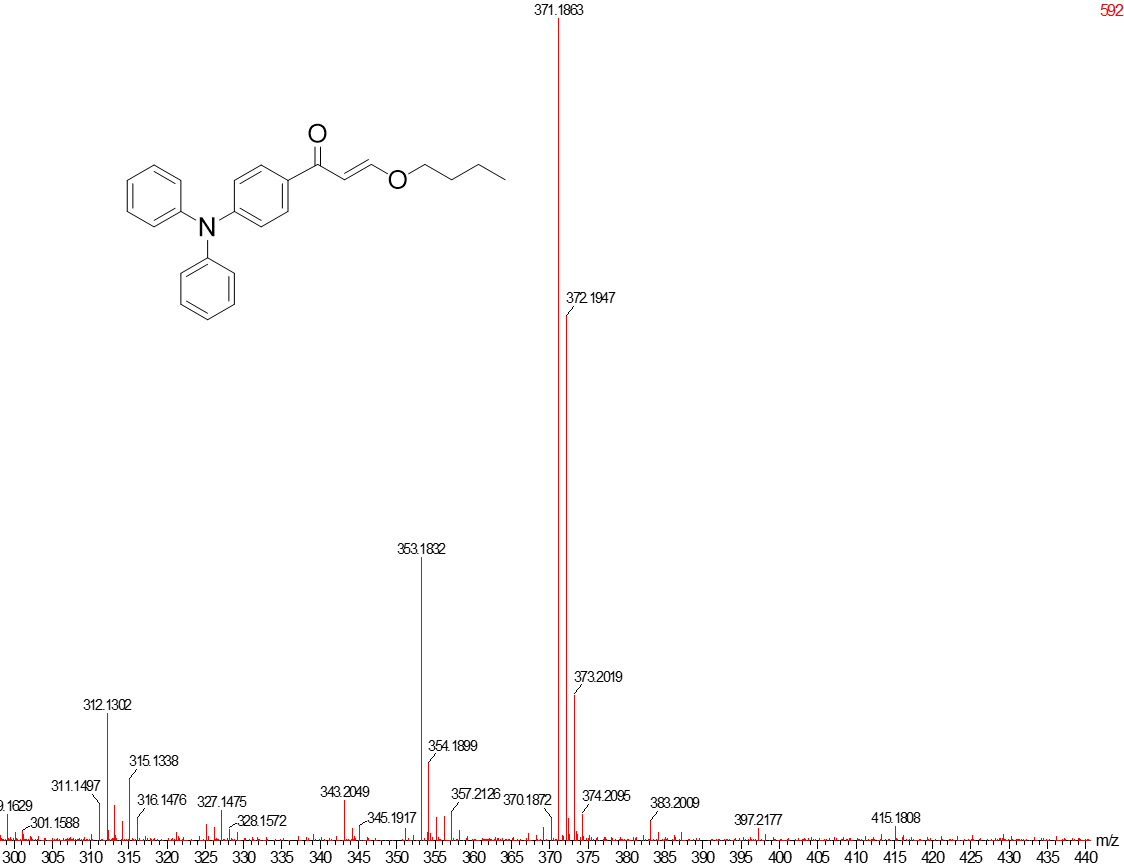


**Figure S18**. HRMS result for the conjugation product between 1-butanol and *alkyne*-TPA.

**Figure S19**. ^1^H-NMR spectra recorded for HPC and the conjugation product of HPC and *alkyne*-TPA, HPC-TPA.

**Figure S20**. FT-IR spectra recorded for the conjugation product of *alkyne*-TPA and HPC, HPC-TPA, respectively.


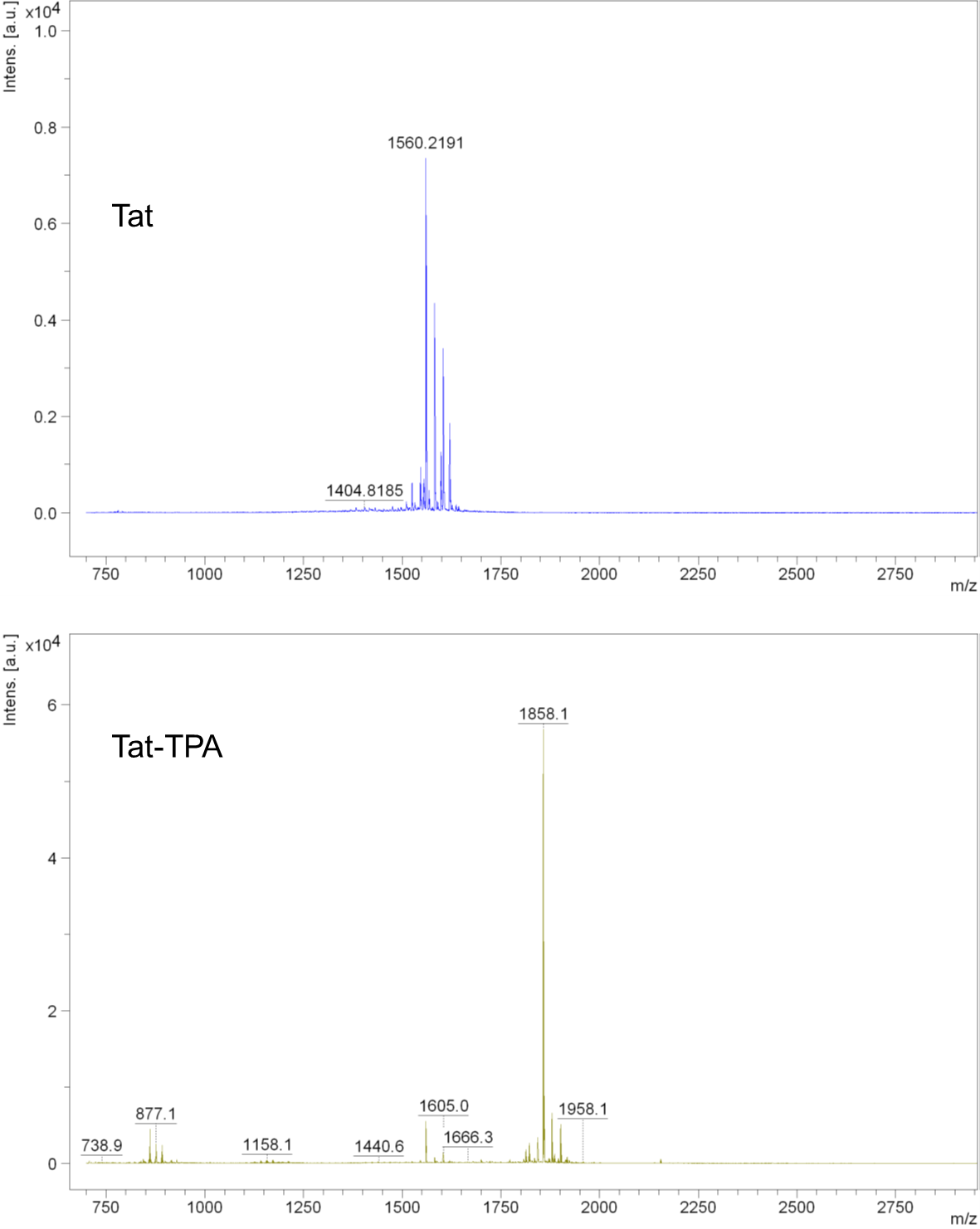


**Figure S21**. MALDI TOF analysis of Tat peptide and *alkyne*-TPA functionalized Tat, Tat-TPA, respectively. The sequence of Tat peptide is YGRKKRRQRRR.


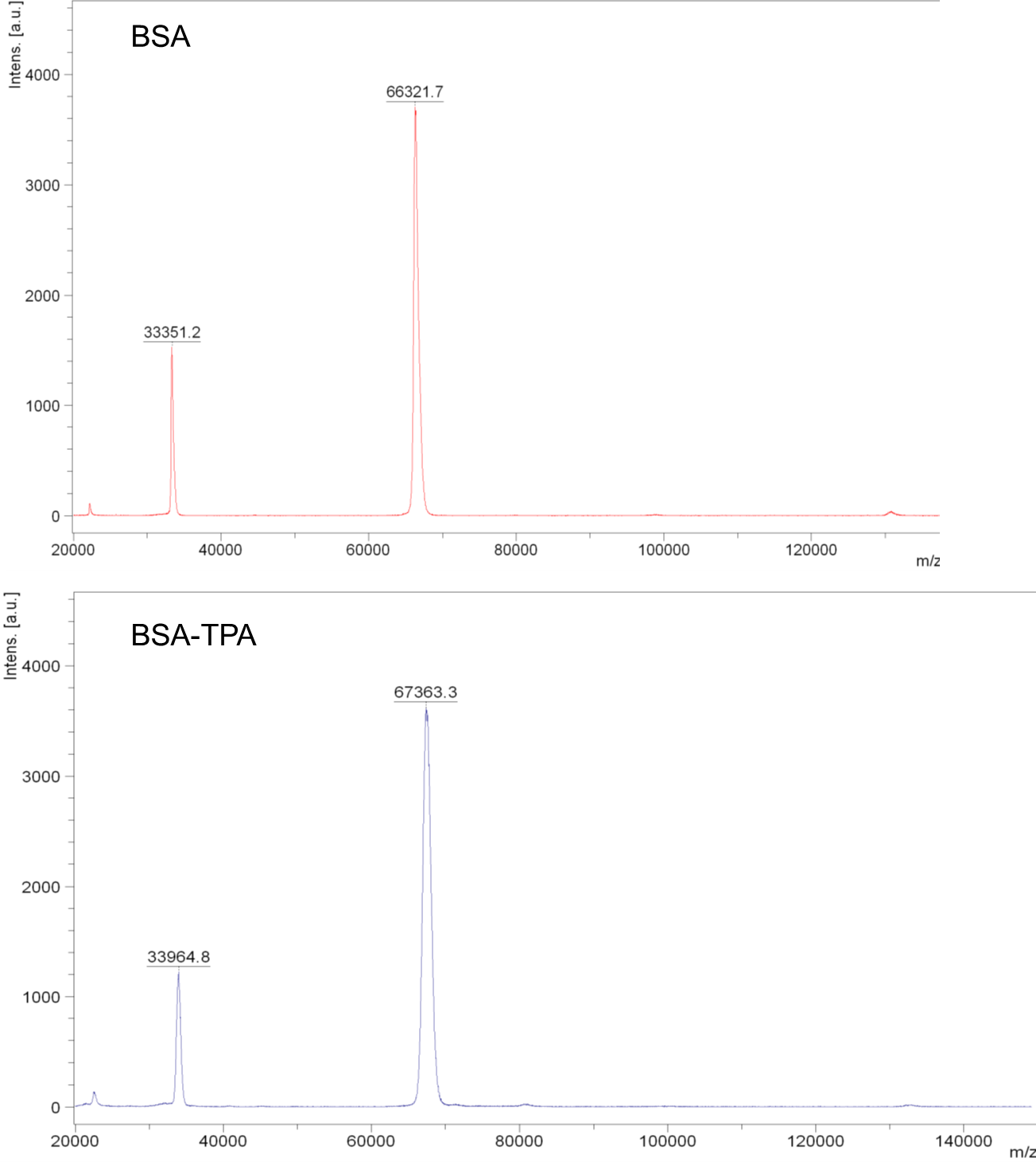


**Figure S22**. MALDI TOF analysis of BSA and *alkyne*-TPA functionalized BSA, BSA-TPA, respectively.


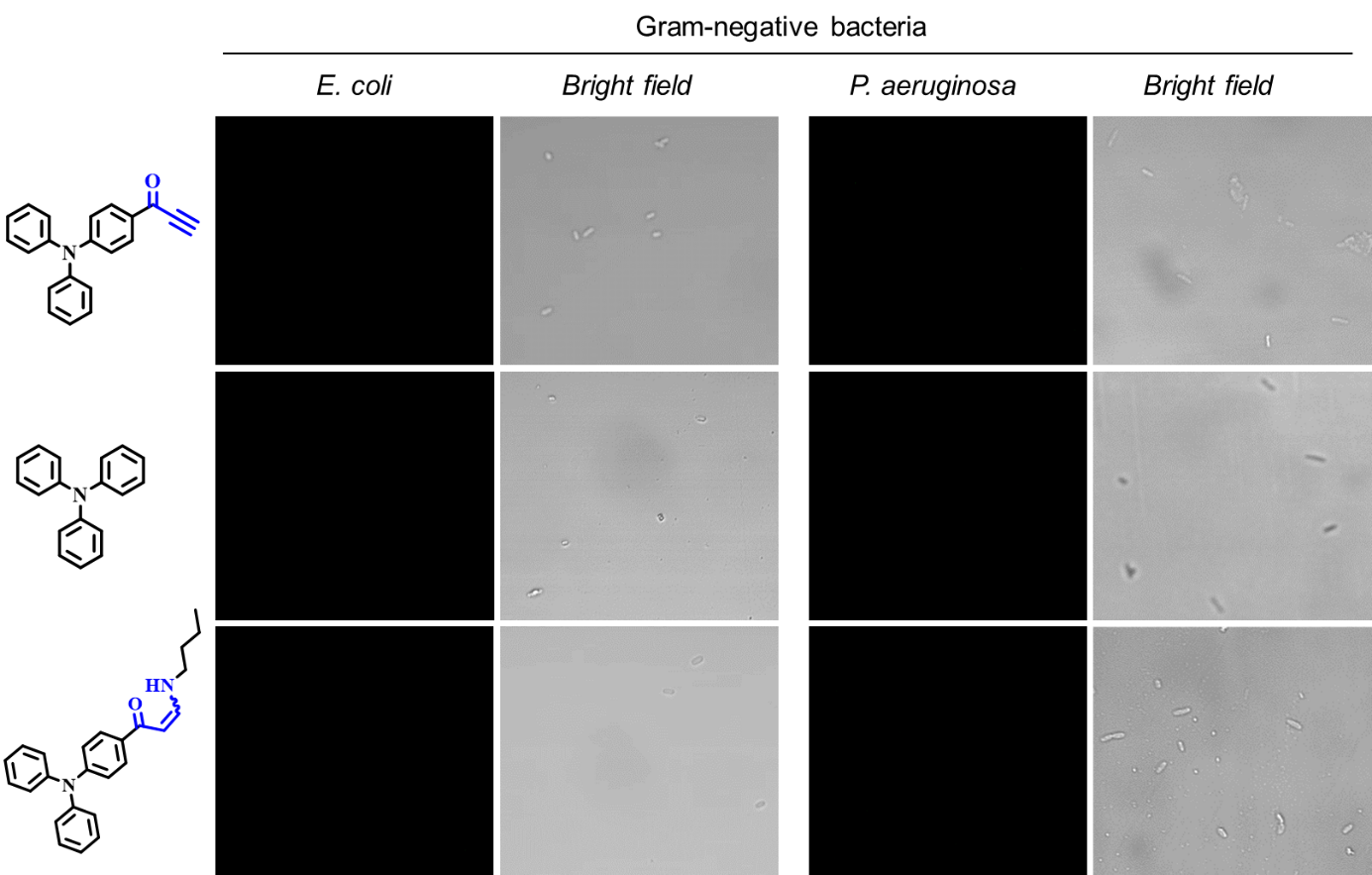


**Figure S23**. Two types of typical Gram-negative bacteria, *Escherichia coli* (*E. coli*) and *Pseudomonas aeruginosa* (*P. aeruginosa*) upon 10 min incubation with three kinds of fluorescent molecules, respectively, including TPA, *alkyne*-TPA, and the addition product of *alkyne*-TPA and n-butylamine.


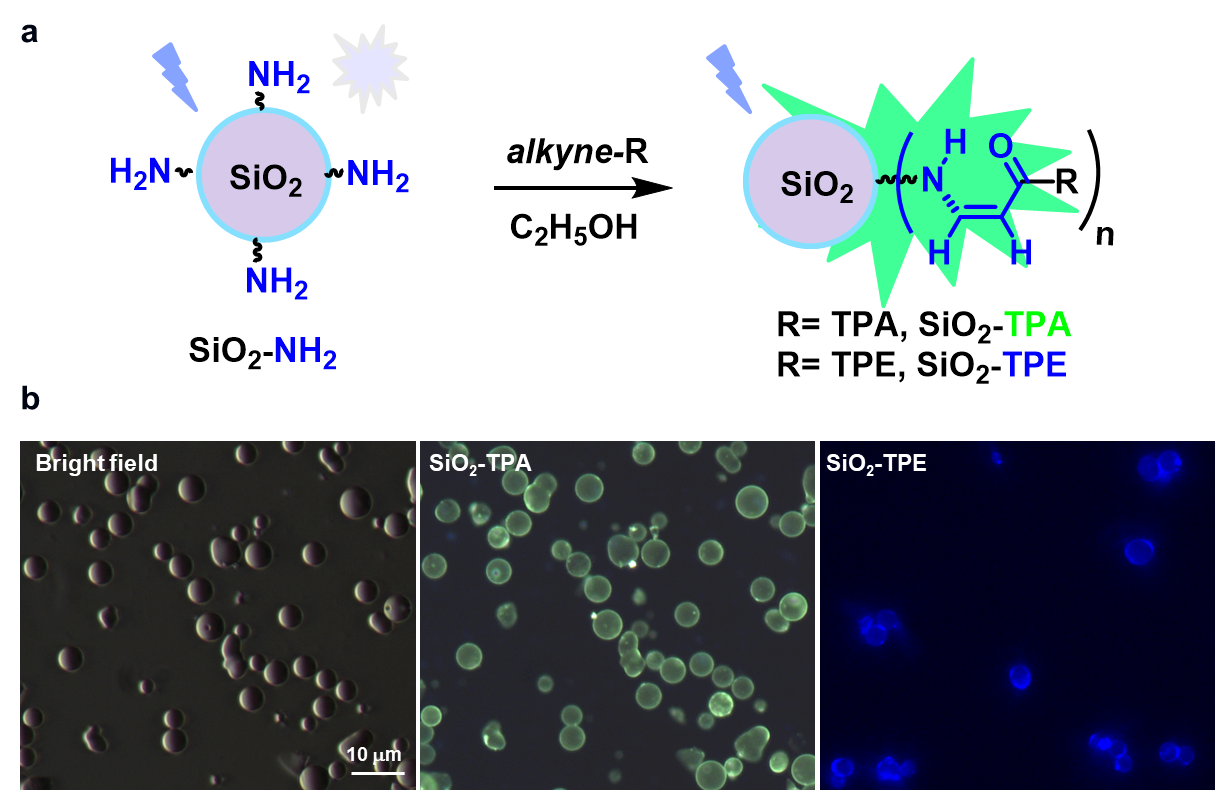


**Figure S24**. Preliminary demonstration for the functionalization of inorganic matrix (such as SiO_2_ particles) via the amino-yne click conjugation. (a) Schematics for catalyst-free fabrication process, affording TPE and TPA-modified SiO_2_ particles, SiO_2_-TPA, SiO_2_-TPE, respectively. (b) Fluorescent microscopy imaging of SiO_2_-TPA, SiO_2_-TPE, respectively (λ_ex_= 365 nm).
